# Cognitive measures predict falls in Parkinson’s disease: Insights from the CYCLE-II cohort

**DOI:** 10.1016/j.parkreldis.2025.107328

**Published:** 2025-02-11

**Authors:** Saar Anis, Eric Zimmerman, A. Elizabeth Jansen, Ryan D. Kaya, Hubert H. Fernandez, Cielita Lopez-Lennon, Leland E. Dibble, Anson B. Rosenfeldt, Jay L. Alberts

**Affiliations:** a Center for Neurological Restoration, Cleveland Clinic, Ohio, USA; b University of Utah, Department of Physical Therapy and Athletic Training, Salt Lake City, UT, USA; c Cleveland Clinic, Department of Biomedical Engineering, Cleveland, OH, USA

**Keywords:** Parkinson’s disease, Falls prediction, Processing speed, Spatial memory, Waiting room of the future

## Abstract

**Background:**

Accurate prediction of falls in patients with Parkinson’s disease (PWP) is crucial for effective prevention efforts. Historically, fall risk models have heavily relied on motor features, overlooking the vital cognitive-motor interplay essential for locomotion.

**Methods:**

Baseline assessments and year-long fall data from the CYClical Lower Extremity Exercise for Parkinson’s disease II (CYCLE-II) trial’s control group were utilized. A LASSO logistic regression model assessed thirty-seven demographic, motor, and cognitive variables to identify key fall predictors. To explore the practical implementation of predicting falls in a clinical setting, a second model was developed using a subset of nine candidate measures conducive for retrieval from electronic medical records. Models’ accuracy was validated against Paul et al.’s 3-step fall prediction model.

**Results:**

Analysis included 123 participants (mean age 65.3 ± 8.3 years, 66 % males, mean disease duration 4.9 ± 4.1 years). Seventy-two participants (58.5 %) fell at least once; with 33.1 % occurring during walking, 34.4 % resulting in injuries. The initial model identified 8 predictors with an AUC of 0.68. The second model, incorporating disease duration and cognitive tests, achieved an AUC of 0.67, comparable to Paul et al.’s validation (AUC 0.66). Participants with poorer information processing and spatial memory were more prone to falling over the 12-month period.

**Conclusions:**

Impaired cognitive performance and longer disease duration were powerful predictors in identifying a future fall in PWP. The link between cognitive performance and potential for falling reinforces the strong interplay between gait and cognition.

## 1. Introduction

Falls represent a significant contributor to morbidity in patients with Parkinson’s disease (PWP). Falls are remarkably prevalent [1], with substantial repercussions such as fractures and hospital admissions [2]. Falls have an adverse psychological dimension as they can induce fear of falling that alter behavior, making them reluctant to engage in physical activity and limit community engagement, thereby negatively affecting health and quality of life [3].

The risk factors for falls in PWP have been well-investigated, encompassing disease-related motor and non-motor determinants, comorbid conditions, and environmental factors [4]. Notably, a history of falls in the preceding year stands out as a strong risk factor [5], followed by gait and balance disorders [6], disease duration [1], fear of falling [7], disease severity [8], and freezing of gait [9]. Importantly, several studies have found global cognition [10] and specific domains such as executive function [11], attention [12] and visuospatial function [10] as suggested risk factors. Several prediction models have been developed based on these known risk factors, exhibiting reasonable accuracy [13]. However, most models face the challenge of conducting numerous assessments, which can be impractical in a busy clinic. Other common limitations are over-reliance on patient recall of fall [5], and the need for instrumented or complicated gait assessments [14].

In recent years, simplified models feasible for clinical settings have emerged to address this complexity. Examples include the "pants sign" [15], which refers to individuals who resort to putting on their pants only while seated, and a three-step model incorporating *falling in the previous year*, *freezing of gait in the past month*, and *comfortable gait speed*, showing promising discrimination (area under curve [AUC] = 0.80) [16]. External validation of the last model has yielded an AUC of 0.74 [17]. Another model that incorporates history of near falls, tandem gait, and retropulsion according to the Nutt Retropulsion Test (NRT), has been proposed [17]. While these efforts are laudable, they primarily focus on motor assessments and lack the inclusion of possible cognitive and non-motor symptoms, both of which occur early and frequently in the disease process [18]. In this era of data collection through wearables and digital tools [19], there is a pressing need to develop automated tools for predicting future falls, that considers a wider spectrum of potential contributors, motor or non-motor, with the primary goal of tailoring prevention measures effectively.

The CYClical Lower Extremity Exercise for Parkinson’s disease II (CYCLE-II) trial [20], was a multi-site randomized controlled trial aimed at assessing the disease-modifying potential of a 12-month, home-based aerobic exercise program in PWP. The study consisted of two groups: exercise intervention and a usual customary care (UCC) group. The project leveraged several distinguished factors: 1) a pragmatic design, enabling evaluation in real-life routine conditions; 2) comprehensive motor and cognitive assessments at baseline; and 3) meticulous data collection regarding falls over a one-year period. The prospectively collected fall data provided an opportunity to effectively address existing gaps in falls analysis and investigate predictive demographic, motor, and non-motor factors for identifying future falls.

The primary aim of the current study was to utilize a comprehensive set of motor and cognitive outcomes to predict falls in a cohort of PWP from the UCC group of the CYCLE-II trial. A secondary aim was to evaluate the effectiveness of the previous 3-step model by Paul et al. [16], which relies on motor and disease-related variables, within the CYCLE-II cohort. The rationale behind this secondary aim was to compare our results to previous models, thereby generalizing and examining the robustness of our models. Our third aim was to evaluate the predictive capacity of a subset of CYCLE-II collected outcomes, specifically those amenable to retrieval from electronic medical records (EMR). The rationale for this aim was to initiate an exploration into a future practical automated approach for risk assessment.

## 2. Methods

### 2.1. Study population

CYCLE-II participants were recruited at two sites, the Cleveland Clinic (Cleveland, OH, USA) and the University of Utah (Salt Lake City, UT, USA). The inclusion and exclusion criteria and data collection methods have been detailed previously [20]. In short, this trial was a single rater, blinded, randomized controlled trial where participants were randomized to a home-based cycling or UCC group. All participants were adults diagnosed with PD according to the UK Brain Bank criteria, scored between I and III on the Hoehn and Yahr (HY) scale, and were on stable doses of antiparkinsonian medications. The exclusion criteria included: cognitive limitations that would impede the patient’s ability to provide consent, history of deep brain stimulation (DBS) or high-intensity focused ultrasound (HIFU) thalamotomy, musculoskeletal issues limiting the ability to exercise, neurological diseases other than PD, and cardiac arrhythmia. All participants completed the informed process prior to participation.

For this project, only data from the UCC group (n = 123) were used. Exercise participants were excluded as the impact of exercise on delaying or preventing falls is unknown [21]. The UCC group was asked to continue their normal level of activity and to not initiate a formal cycling program during study participation. Referrals to physical, occupational, and speech therapies were not restricted, as they reflect standard of care in PD.

### 2.2. Demographic and clinical data collection

As part of the study protocol, participants underwent a comprehensive enrollment assessment. This assessment included collecting demographic and medical data, such as age, sex, years since diagnosis, more affected side, education level, and recall data regarding number of falls within the previous 6 months. A medication reconciliation was conducted, and the Levodopa Equivalent Daily Dose (LEDD) was calculated. Participants also underwent a series of motor assessments: Movement Disorder Society-Unified Parkinson’s Disease Rating Scale (MDS-UPDRS) part III, the Manual Dexterity Test (MDT) to evaluate skilled hand function [22], the Instrumented Timed Up and Go (TUG) test, the 6-min Walk Test (SMWT), comfortable and fast walking speed via the 10 m walk, and assessments of postural stability under two stances (feet together-eyes closed and tandem stance-eyes open). Cognitive assessments included: the Processing Speed Test (PST) [23], Trail Making Test (TMT) parts A and B, and Spatial Memory Test (SMT) [24] for evaluating episodic and delayed memory. Participants also completed patient-reported assessments of Neuro Quality of Life (Neuro-QoL) in the domains of upper and lower extremity function. Finally, MDS-UPDRS parts I, II, and IV were collected. Assessments were conducted in the “off-medication” state, practically defined as withholding antiparkinsonian medications for a minimum of 12-h prior to the assessment.

Throughout their 12-month participation, detailed fall data were prospectively obtained and confirmed during a bi-weekly telephone call. Each participant was provided with a fall diary in which each fall with corresponding information about the environment and circumstances was recorded. Specifically, a 50-week study duration was analyzed to account for the ± 10-day visit window for the 12-month assessment. During the first two months of the study, falls were documented, but their circumstances and characteristics were not recorded, resulting in approximately 13 % of participants experiencing at least one fall without this information.

### 2.3. Statistical analysis

Participants were categorized as a faller (1 or more falls [17]), or non-faller (0 falls) based on the number of falls observed over the 50-week period. Differences in baseline outcomes between fall groups were assessed using Welch’s t-tests for approximately normally distributed outcomes, Wilcoxon rank sum tests for skewed numeric outcomes, and chi-squared tests for categorical outcomes. Given that four individuals experienced a disproportionate number of falls (falls ranging from 71 to 874 over 50-weeks), fall characteristics were summarized as the mean sample proportion, so participants were equally weighted. For each participant, their individual proportion for each characteristic of falls was calculated, and then proportions were averaged across all participants.

To investigate the predictability of falls, two Least Absolute Shrinkage and Selection Operator (LASSO) logistic regression models were created [25]. The first model utilized demographic, motor and non-motor outcomes obtained from the baseline off medication visit, encompassing a total of 37 measures (detailed in Supplementary Table 1). This model aimed to forecast the probability of an individual experiencing a fall over the subsequent year. Expanding upon established prediction tools such as the 3-step falls prediction model for PWP [16], the MDS-UPDRS III question 3.11 (freezing of gait) and MDS-UPDRS part II question 2.13 related to the frequency of freezing were included as additional separate variables.

The second model focused on a narrower subset of 9 variables from the initial pool of 37. This subset was selected to include measures that are amenable to retrieve from EMRs, including MDS-UPDRS part 3 (both total score and question 3.11), years since diagnosis, sex, and age. Four other variables - SMT total correct, PST total correct, comfortable walking average speed (m/s), and MDT average duration (s) - are routinely collected at the Cleveland Clinic’s Center for Neurological Restoration as part of the ‘Waiting Room of the Future’ initiative, which has been gathering these data for all Parkinson’s disease patients prior to their physician visits since 2021 [26]. The iPad-based tests used in this initiative were also administered as part of the CYCLE-II study outcomes.

The predictive capability of the LASSO logistic regression models was determined using 5-fold cross-validation. Five times, a model was created on the training subsets containing 80 % of the data. To determine the optimal lambda parameter, each training subset was further divided with nested cross-validation. Models were constructed with a range of 100 possible lambda parameters, and the best performing lambda by binomial deviance was used to construct the model for each training subset. Each model was then tested on the corresponding test data sets, and classification capability was assessed using receiver operating characteristic (ROC) curves and the average AUC. The overall LASSO logistic regression models were then created from the entire dataset, with the optimal lambda parameter again determined using cross-validation. Reported coefficients have been back transformed from the log-scale to odds ratios of being a faller over not being a faller.

Validation of the three-step model by Paul et al. [16] was done using logistic regression with three predictors: the *presence of any self-reported falls over the previous six months from baseline*, the *presence of any freezing of gait over the previous week*, and whether *comfortable gait speed was less than 1.*1 m/s *or not*. Although the original three-step model included the presence of falls over the previous 12 months and freezing of gait over the previous month, a shorter date range was used in the validation due to availability of the data. Because the three-step model was built to predict the presence of falls during the following six months from baseline, it was first validated to predict six-month faller status. Secondly, to compare to the LASSO logistic models, the three-step model was then used to predict 12-month falls. As before, the three-step model was trained and tested using five-fold cross-validation.

All statistical analyses were conducted using RStudio version 2023.06.01, R version 4.2.3.

## 3. Results

### 3.1. Study population and baseline clinical assessments

From the CYCLE-II trial, data from a total of 123 PWP who experienced a total of 1521 falls during 1-year of follow-up informed the model. Participants had an average age of 65.3 ± 8.3 years, 65.9 % males, and an average disease duration of 4.9 ± 4.1 years. Demographic, motor, cognitive, and quality of life measures at baseline are detailed in Table 1. During the 1-year follow-up period, 72 patients (58.5 %) experienced falls, while 51 (41.5 %) remained non-fallers. Among the fallers, 32 (44.4 %) reported falls prior to study enrollment, and 40 (55.5 %) were new fallers. Among non-fallers, 9 (17.6 %) reported previous falls. The corresponding data for each group is also included in Table 1 with significant differences between future fallers and nonfallers in baseline variables bolded. Differences between the two groups included fallers having a longer disease duration, higher LEDD, and worse scores in MDS-UPDRS parts II, III, and IV. Furthermore, fallers exhibited a shorter distance covered during the 6-min walk test, and lower NQoL upper- and lower-extremity T-Scores. From a cognitive perspective, future fallers needed more time to complete TMT part B and achieved lower scores on the PST. Lastly, the future fallers reported significantly more falls 6 months prior to study entry.

### 3.2. Falls data during one year of follow-up

Fig. S1 depicts the number of falls for each participant included in the modeling. Table 2 provides data related to falls within our cohort (presented as proportions), documenting 1521 falls during 1 year of follow-up. The primary activities during falls were walking (33.1 %), followed by engaging in outdoor activities (22.0 %), and stairs/curb (15.3 %). The distribution of falls showed a near-identical split, with indoor falls constituting 49.5 % and outdoor falls comprising 50.5 % of the total incidents. Most falls (65.3 %) happened during forward movement. Approximately 35 % of falls resulted in injuries, and 13.1 % of all falls required formal follow-up medical care. Regarding the type of injury, soft tissue injuries were the most prevalent (32.4 % of falls) followed by bone fractures (7.2 % of falls).

### 3.3. Identifying predictors for future falls within the next year

**Fig. 1.**
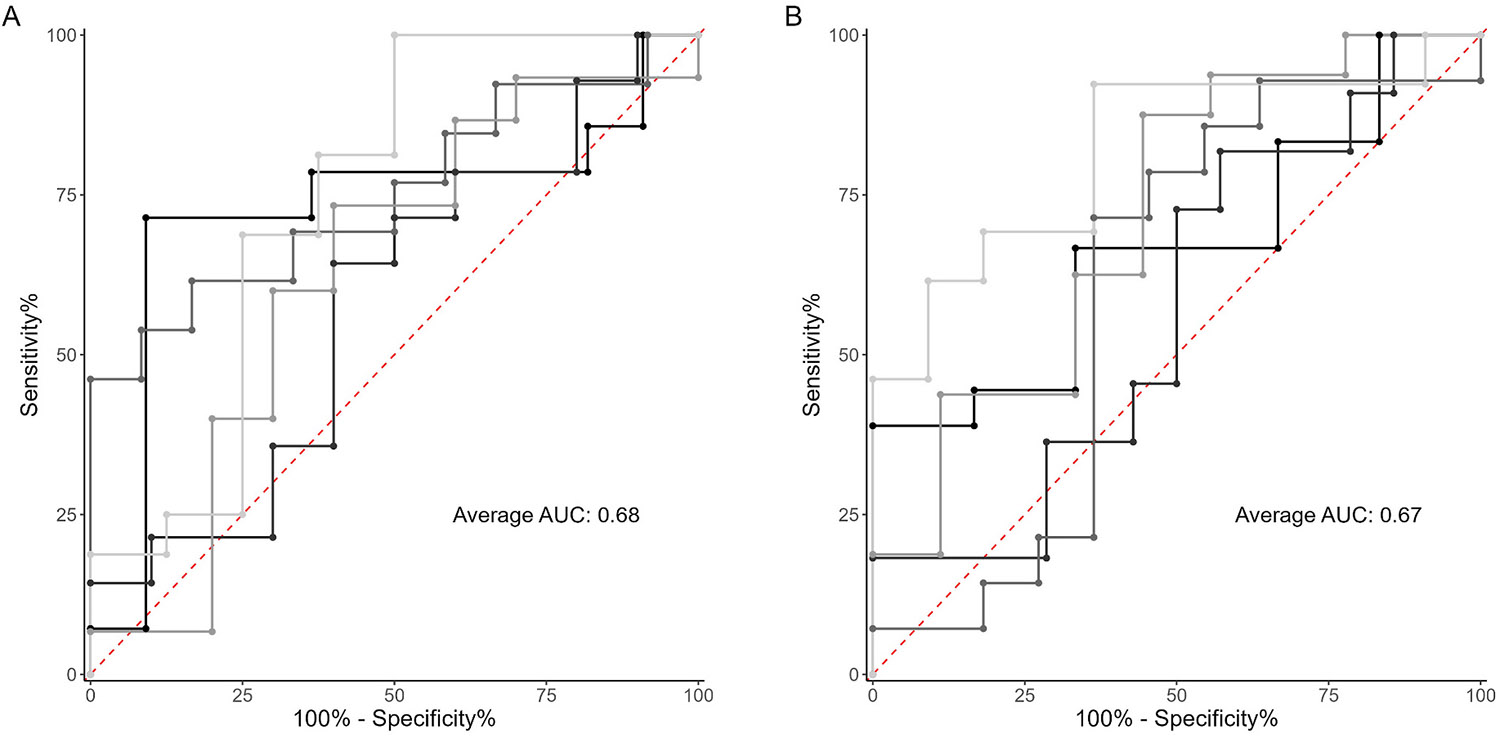
Predictive performance of LASSO logistic regression models. **A** – An ROC curve, depicting the results from the 5 cross validated models, built off all candidate predictors. **B** – An ROC curve, depicting the results from the 5 cross validated models, built off *EMR-retrievable* predictors.

Thirty-seven candidate baseline demographic, motor, and non-motor measures were employed in the model (for the full list, refer to Supplementary Table 1). The final output consisted of 8 measures, including number correct in PST (coefficient = 0.989), NQoL upper extremity T-score (0.994), SMT time (1.001), MDS-UPDRS part II total (1.063), SMWT distance (0.999), question 2.13 on MDS-UPDRS part II (1.016), years since diagnosis (1.093), and previous one or more falls within 6 months prior to study recruitment (1.247). These 8 measures yielded an average (SD) AUC prediction of 0.68 (0.08), as depicted in Fig. 1 A.

### 3.4. Validation of the three-step model (Paul et al., 2013) within our cohort

The three-step model predicting the presence of falls over a six-month period achieved an average (SD) AUC of 0.69 (0.10). When predicting fallers over twelve months, the model achieved an average (SD) AUC of 0.66 (0.10).

### 3.5. Identifying predictors from a narrowed subset of potentially EMR-retrievable measures

**Fig. 2.**
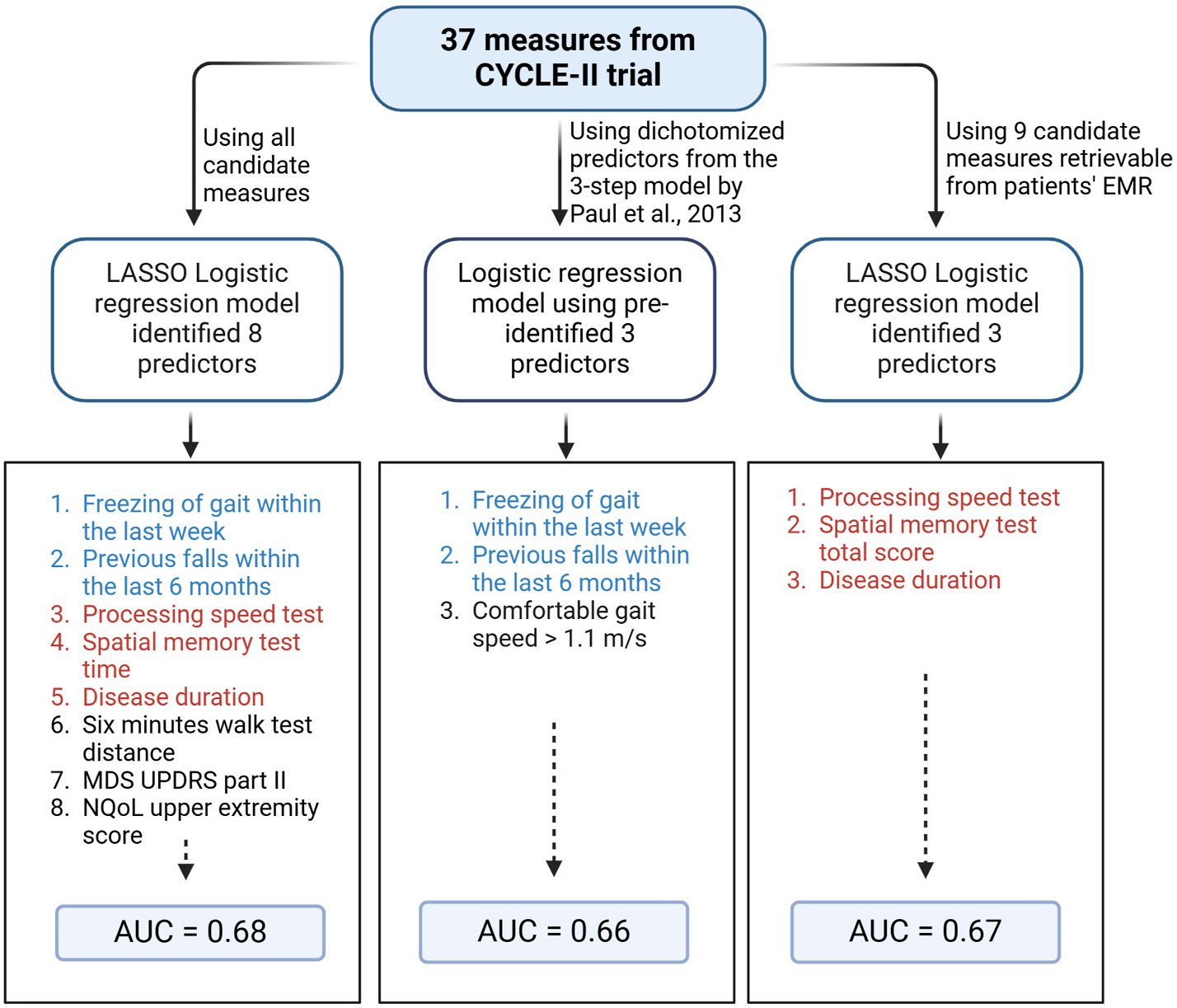
A comparison of the accuracy of our two final LASSO logistic regression models (left and right) and the validation of the 3-step model by Paul et al. (2013) (middle). Red text highlights similar metrics between the LASSO models, while blue text indicates similarities between the first LASSO model and the 3-step model.

Our final aim was to develop a model that could be applied to data accessible from patients’ EMRs. In pursuit of this goal, a subset of 9 measures were assessed (indicated with an asterisk in Supplementary Table 1). The model identified 3 predictive measures: PST total correct (coefficient = 0.970), SMT total correct (0.998), and years since diagnosis (1.144). The model achieved an average (SD) AUC of 0.67 (0.10), as shown in Fig. 1 B. The predictor odds ratio against the lambda tuning parameter are provided in Fig. S2. Fig. 2 demonstrates the summary results of all identified and validated models.

## 4. Discussion

In this project, motor and cognitive outcomes from the control group of the CYCLE-II randomized clinical trial were evaluated in their capability to predict future falls in PWP. Primary findings include the development of an 8-outcome predictive model for future falls utilizing both motor and cognitive data. Furthermore, we have demonstrated that a set of 3 EMR extractable outcomes possess a comparable predictive ability without including any direct motor outcome. These findings hold potential for real-time alert systems that could notify providers about the risk of falls, facilitating timely intervention. Moreover, the comparable performance of the developed predictive models to existing ones underscores their relevance and potential applicability in clinical settings.

Aligning with previous research [15,27], future fallers had significantly longer disease duration and higher scores in MDS-UPDRS parts II-IV, indicating disease burden is a key contributor to balance decline and fall risk. Contrary to findings in prior studies and meta-analyses [28,29] most baseline gait parameters and balance assessments (e.g., TUG, gait speed and static balance) were not statistically different between future fallers and non-fallers, which most likely contributed to their exclusion from the final predictive models.

Prior research has shown that global cognitive performance [7] and specific cognitive domains, such as attention and executive function, are linked to gait slowing, instability, and future falls in PD [30]. Both of our models revealed that both information processing speed and spatial memory emerged as strong predictors for future falls. The association of slow processing speed with fall prediction aligns with previous findings in older adults with a history of falls [31], supporting our findings. Processing speed dysfunction may contribute to falls due to its impact on various cognitive processes. When individuals experience delays in processing information, their reaction time to respond appropriately to environmental stimuli is slowed [32]. This slowing can result in difficulty adjusting to changes in terrain or obstacles, increasing the likelihood of falling [33]. Moreover, slowed processing speed may lead to challenges in attending to the visual, somatosensory, and auditory information necessary for safe community ambulation [34]. Impaired attentional control associated with processing speed dysfunction may lead to decreased awareness of potential hazards in the environment [35]. Cognitive overload, such as what occurs during community ambulation, can overwhelm PWP, further compromising their ability to maintain balance and prevent falls [36]. Moreover, compromised executive function, which often accompanies processing speed dysfunction [37], impairs decision-making and planning abilities, making it difficult for individuals to anticipate and avoid fall-inducing situations [38].

While it may not be intuitive that spatial memory impacts falls, this relationship could be mediated by attention [39]. Specifically, spatial attention has been found to influence gait difficulties and falls [10]. Alternatively, it could arise from the interconnected nature of spatial short-term memory and working memory, both relying on executive resources and not being entirely distinct [40], and dysfunction in these aspects might serve as a surrogate for executive dysfunction. However, additional investigation is necessary to delineate the specific contributions of spatial memory to falling.

Importantly, MDS-UPDRS part II, capturing motor experiences of daily living, played a prominent role in our final 8-measure model, underscoring its importance in fall prediction. Distinguishing itself from part III, often employed as a primary outcome measure for disease severity and progression, the subjectively reported part II provides insights into the impact of PD on routine activities reported by the patient and/or caregiver. A recent publication further supports this notion, revealing a correlation between questions 11–13 from MDS-UPDRS part II (which address getting out of bed, car, or deep chair, walking and balance, and freezing), and postural instability, along with their predictive value for future falls [41]. This underscores the importance of considering this measure, or its specific components, in future studies seeking predictors for falling.

Our best 8-measure model incorporated 2 out of 3 measures from the 3-step simple model by Paul et al. [16] with comparable efficacy. However, our external validation of the Paul et al. model revealed lower efficacy compared to previous validation [17], or the original cohort. This is partly attributable to our use of cross-validation, where the predictive capability of the model is tested on data not used to train it, reducing the chance of overfit. Additionally, slight variations in predictor definitions might have played a role; for instance, we considered recall falls (yes/no) in the previous 6 months and freezing of gait in the previous week, as opposed to 1 year and 1 month respectively in the original model creation.

An important implication from this project is the predictive capability of data retrievable from EMR. In recent years, the Cleveland Clinic has been pioneering the PD ’Waiting Room of the Future’ (WROTF) [26], which entails gathering self-administered iPad-based assessments of motor and cognitive functions from PWP in the waiting room. Outcomes are automatically calculated and integrated into the EMR prior to their in-person visit with the healthcare provider. Many of the same iPad-based tests used in the CYCLE-II trial are also gathered as part of routine care in the WROTF. The predictive ability of processing speed and spatial memory is pivotal in our pursuit of identifying at risk patients and providing real-time notifications to their healthcare providers. Subsequently, the informed provider can implement timely interventions to prevent falls, either individually or through collaboration with a multidisciplinary team and appropriate referrals to clinical specialists such as PD trained physical therapists. Further real-world investigation, involving the continued integration of cognitive testing and monitoring results concerning falls, followed by monitoring the effects of early intervention in a highly pragmatic manner, is essential to validate this concept. If successful, it holds promise for advancing personalized medicine by seamlessly integrating technology into the waiting room. A key strength of our model is that it predicts falls without relying on prior falls as a predictor, making it particularly valuable for identifying individuals at risk before their first fall. By focusing on cognitive measures and disease duration, our model offers a proactive and forward-looking approach.

While the current study provides insights into the value of using cognitive outcomes to predict falls, it also points towards several avenues for future research. Future models could incorporate an analysis of near falls, and subsequent assessments could differentiate between onetime and recurrent fallers. Additionally, our predefined variables for assessing gait, balance and cognition were partial, and alternative motor outcome measures, such as the Balance Evaluation Systems Test (BEST) and its variants (e.g., the mini-BEST), which have been found as good indicators for future fall in PD [42], might have yielded different conclusions. Furthermore, incorporating additional variables such as environmental factors and pharmaceutical interactions could provide a more comprehensive risk profile, improving the model’s predictive power and clinical applicability. Other studies examining broader cognitive profiles in PD patients have reported conflicting results compared to ours. They found that fallers exhibited poorer performance in executive function and attention compared to non-fallers, but similar performance in memory and information processing, which contradicts our findings [43]. The differences in findings between our study and others regarding cognitive profiles in PD patients suggest that there may be variability in how cognitive functions relate to fall risk within different patient populations or study contexts. An important study limitation is the lack of assessment in the visuospatial domain. Therefore, future research should consider these factors and explore more comprehensive cognitive assessments to better understand the relationship between cognitive function and fall risk in PD. Another consideration for future studies is the duration of follow-up. Longer follow-up periods would provide greater insights into how fall risk evolves over time and help identify predictors that may emerge later in the disease trajectory. Last, while our study’s sample was sourced from two locations, Utah and Ohio, which strengthens the robustness of our findings, replication in a larger, more diverse population will be important to ascertain the generalizability of the predictive model across different demographic and clinical circumstances.

In summary, our study’s main strengths lie in the extensive dataset collected from a large population with an appropriate long-term monitoring and follow-up period. Frequent monitoring over the course of one year facilitated the identification of important factors associated with falls and formulate a set of variables predicting future falls, encompassing demographic, motor, and cognitive aspects of function. While balance and gait measures play a crucial role in predicting falls, our findings underscore the importance of cognitive impairment as another essential contributor to the evolving fall identification and management algorithm for PWP.

## Supplementary Material

Supplementary Figure 2

Supplementary Table 1

Supplementary Figure 1

## Figures and Tables

**Table 1:** Demographic, motor, cognitive, and quality of life measures at baseline for the study cohort.

Non- Faller (N = 51)	Faller (N = 72)	Overall (N = 123)	Effect Size	P-value
**Site**					0.278^b^
Cleveland Clinic	30 (58.8 %)	34 (47.2 %)	64 (52.0 %)		
University of Utah	21 (41.2 %)	38 (52.8 %)	59 (48.0 %)		
**Age, years**					
Mean (SD)	64.1 (7.84)	66.1 (8.59)	65.3 (8.31)	0.23	0.195^a^
**Sex**				0.06	0.675^b^
Female	19 (37.3 %)	23 (31.9 %)	42 (34.1 %)		
Male	32 (62.7 %)	49 (68.1 %)	81 (65.9 %)		
**Years since diagnosis**					
Mean (SD)	3.37 (2.32)	6.00 (4.71)	4.91 (4.10)	0.67	**8.4e-05** ^a^
**LEDD**					
Mean (SD)	611 (339)	760 (385)	698 (373)	0.4	**0.0256** ^a^
**MDS-UPDRS part 1, total score**					
Mean (SD)	6.98 (4.72)	8.32 (4.96)	7.76 (4.88)	0.27	0.132^a^
**MDS-UPDRS part 2, total score**					
Mean (SD)	5.78 (4.15)	9.47 (5.75)	7.94 (5.44)	0.71	**6.7e-05** ^a^
**MDS-UPDRS part 2, question 13 (freezing)**					
Mean (SD)	0.10 (0.30)	0.47 (0.87)	0.32 (0.72)	0.54	**0.00109** ^a^
**MDS-UPDRS part 3, total score**					
Mean (SD)	33.71 (13.44)	38.76 (14.21)	36.67 (14.07)	0.36	**0.0471** ^a^
**MDS-UPDRS part 3, question 11 (freezing)**					
Mean (SD)	0.47 (0.78)	0.54 (0.85)	0.51 (0.82)	0.09	0.634^a^
**MDS-UPDRS part 4, total score**					
Mean (SD)	2.00 [0, 11.0]	4.00 [0, 12.0]	3.00 [0, 12.0]	0.39	**0.0296** ^a^
**Prior falls within the previous 6 months**				0.28	**0.0036** ^b^
Yes	9 (17.6 %)	32 (44.4 %)	41 (33.3 %)		
No	42 (82.4 %)	40 (55.6 %)	82 (66.7 %)		
**MDT trial average duration**					
Mean (SD)	27.5 (9.92)	27.0 (4.47)	27.2 (7.21)	0.06	0.753^a^
**TUG average velocity during turning,** $\left(\frac{deg}{s}\right)$					
Mean (SD)	81.8 (16.6)	76.7 (16.4)	78.8 (16.6)	0.3	0.0984^a^
**TUG average duration, seconds**					
Mean (SD)	10.2 (2.44)	10.4 (3.04)	10.3 (2.80)	0.09	0.617^a^
**TUG average peak velocity during turning,** $\left(\frac{deg}{s}\right)$					
Mean (SD)	144 (37.6)	141 (38.2)	142 (37.8)	0.08	0.659^a^
**TUG average turn duration, seconds**					
Mean (SD)	2.16 (0.457)	2.34 (0.701)	2.26 (0.615)	0.28	0.102^a^
**Comfortable walking average velocity,** $\left(\frac{m}{s}\right)$					
Mean (SD)	1.29 (0.217)	1.22 (0.213)	1.25 (0.217)	0.32	0.0857^a^
**Fast walking average velocity,** $\left(\frac{m}{s}\right)$					
Mean (SD)	1.74 (0.332)	1.63 (0.320)	1.68 (0.328)	0.33	0.0763^a^
**Six minutes’ walk test distance, meters**					
Mean (SD)	505 (82.4)	447 (126)	471 (113)	0.52	**0.0025** ^a^
**Feet together, eyes closed volume,** ${\left(\frac{m}{s}\right)}^{2}⋆{\left(\frac{m}{s}\right)}^{2}⋆{\left(\frac{deg}{s}\right)}^{2}$					
Median [Min, Max]	0.5 [0.04, 10.8]	0.47 [0.05, 30.86]	0.49 [0.04, 30.86]	0.2	0.803^c^
**Tandem stance volume,** ${\left(\frac{m}{s}\right)}^{2}⋆{\left(\frac{m}{s}\right)}^{2}⋆{\left(\frac{deg}{s}\right)}^{2}$					
Median [Min, Max]	1.83 [0.31, 562.9]	3.38 [0.35, 334.39]	2.92 [0.31, 562.9]	0	0.105^c^
**Feet together, eyes closed area,** ${\left(\frac{m}{s}\right)}^{2}⋆{\left(\frac{m}{s}\right)}^{2}$					
Median [Min, Max]	0.02 [0, 0.14]	0.02 [0, 0.3]	0.02 [0, 0.3]	0.2	0.689^c^
**Tandem stance area,** ${\left(\frac{m}{s}\right)}^{2}⋆{\left(\frac{m}{s}\right)}^{2}$					
Median [Min, Max]	0.04 [0.01, 2.15]	0.06 [0.01, 1.77]	0.05 [0.01, 2.15]	0.04	0.103^c^
**Trail making test Part A time, seconds**					
Mean (SD)	31.5 (13.5)	34.5 (14.3)	33.2 (14.0)	0.21	0.236^a^
**Trail making test Part B time, seconds**					
Mean (SD)	49.1 (23.2)	64.3 (40.2)	58.0 (34.9)	0.44	**0.00919**^a^
**PST, number correct**					
Mean (SD)	46.2 (9.57)	41.2 (10.3)	43.2 (10.3)	0.5	**0.00649**^a^
**SMT time, seconds**					
Mean (SD)	127 (58.0)	146 (51.3)	138 (54.7)	0.34	0.0738^a^
**SMT Delayed Recall Time, seconds**					
Mean (SD)	23.1 (9.53)	25.6 (9.44)	24.6 (9.52)	0.26	0.156^a^
**SMT, total correct**					
Mean (SD)	48.9 (13.5)	44.5 (15.0)	46.3 (14.5)	0.31	0.086^a^
**SMT Delayed Recall, total score**					
Mean (SD)	10.5 (3.66)	10.2 (3.36)	10.3 (3.47)	0.08	0.661^a^
**NQoL Upper Extremity, T-Score**					
Mean (SD)	43.4 (8.35)	39.6 (5.58)	41.2 (7.10)	0.56	**0.00513**^a^
**NQoL Lower Extremity, T-Score**					
Mean (SD)	50.6 (6.88)	46.7 (7.11)	48.3 (7.24)	0.55	**0.00288**^a^

Bold indicates measures with a p-value <0.05.

Abbreviations: LEDD, levodopa equivalent daily dose; PD, Parkinson disease; UPDRS, unified Parkinson’s disease rating scale; MDT, manual dexterity test; PST, processing speed time; SMT, spatial memory test; TUG, timed up and go.

a Welch’s Two Sample *t*-test.

b Chi-Square test.

c Wilcoxon Rank Sum test.

**Table 2:** Falls data during one year of follow-up.

Metric	Proportion of documented falls
**Activities during falls**	
Walking	0.331
Performing outdoor activities	0.220
Stairs/Curb (indoor or outdoor)	0.153
Performing household activities	0.127
Transferring (i.e. sit to/from stand)	0.094
Exercise	0.034
Other	0.033
Getting in/out of the car	0.008
Showering or bathing	0.003
**Circumstances of falls** ^a^	
Indoor	0.505
Outdoor	0.495
Dual task	0.183
Freezing of gait	0.093
**Characterization of falls** ^a^	
Moving forward	0.653
Trip	0.281
Slip	0.143
Moving backward	0.083
Turning	0.044
None of the above	0.066
Lightheadedness	0.022
**Falls followed by any type of injury Specific injury** ^a^	0.344
Soft tissue injury	0.324
Bone fracture	0.072
Internal head injury	0.000
Other	0.001

a The sum of the results may exceed 1 because multiple conditions could be assigned for each of the questions.

## Data Availability

The data analyzed in this study is subject to the following licenses/restrictions: The data were sourced from the CYCLE-II study database. The original contributions presented in the study are included in the article, further inquiries can be directed to the corresponding author. Requests to access these datasets should be directed to Jay L. Alberts, PhD, at albertj@ccf.org.

## Appendix A. Supplementary data

Supplementary data to this article can be found online at https://doi.org/10.1016/j.parkreldis.2025.107328.
