# Supplementary figures and images for: Cognitive measures predict falls in Parkinson’s disease: Insights from the CYCLE-II cohort
Source: Parkinsonism Relat Disord. Author manuscript; Available in PMC 2026 Mar 17. (PMC12993869; doi:10.1016/j.parkreldis.2025.107328)

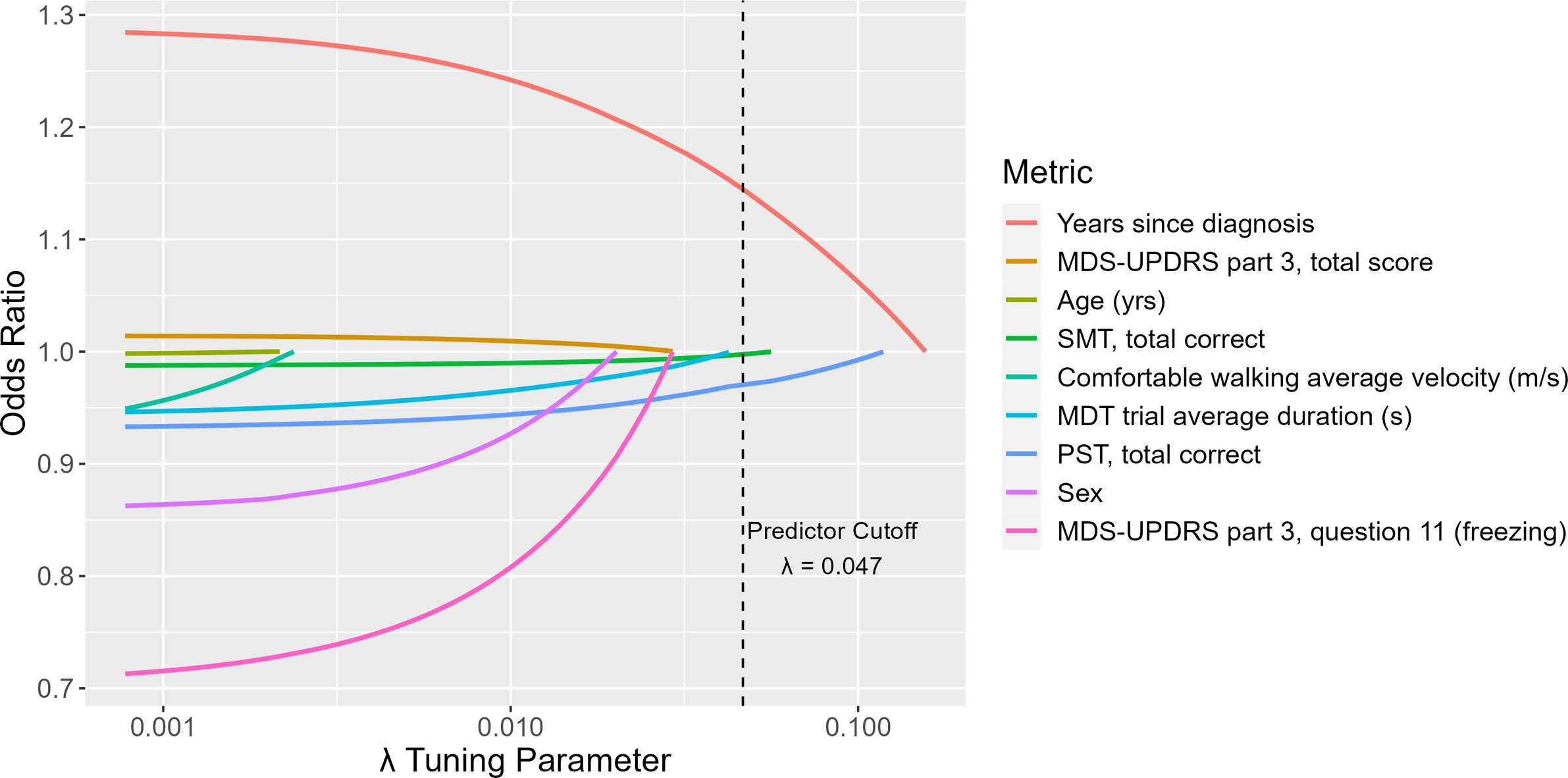

Supplement: Supplementary Figure 2 [file NIHMS2148493-supplement-Supplementary_Figure_2.jpg]

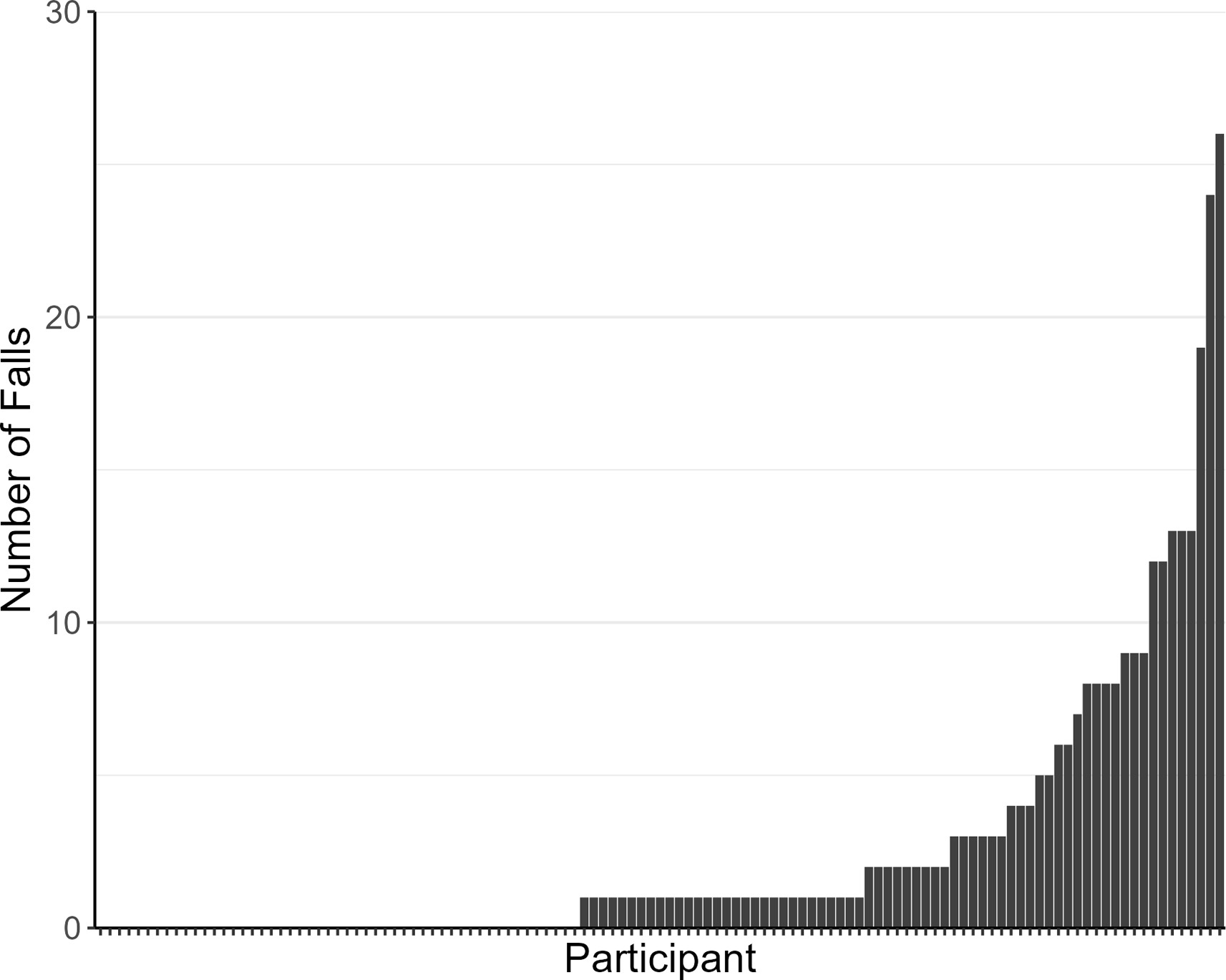

Supplement: Supplementary Figure 1 [file NIHMS2148493-supplement-Supplementary_Figure_1.jpg]
