# Supplementary material for: Cognitive measures predict falls in Parkinson’s disease: Insights from the CYCLE-II cohort
Source: Parkinsonism Relat Disord. Author manuscript; Available in PMC 2026 Mar 17. (PMC12993869; doi:10.1016/j.parkreldis.2025.107328)
Supplement: Supplementary Table 1 [file NIHMS2148493-supplement-Supplementary_Table_1.docx]

Supplementary Table 1. Candidate measures incorporated within the LASSO model to predict future occurrences of falls.

| - | Measure (unit) |
| --- | --- |
| 1 | TMT-A, total time (s) |
| 2 | TMT-B, total time (s) |
| 3 | PST, number correct* |
| 4 | NQoL upper extremity, T-score |
| 5 | NQoL lower extremity, T-score |
| 6 | NQoL fatigue, T-score |
| 7 | NQoL positive affect well-being, T-score |
| 8 | NQoL ability to participate in social roles, T-score |
| 9 | SMT time (s) |
| 10 | SMT delayed recall time (s) |
| 11 | SMT, total correct* |
| 12 | SMT delayed recall, total score |
| 13 | BESS double leg volume ${\boldsymbol{((}\frac{m}{s})}^{2}*\left( \frac{m}{s} \right)^{2}*\left( \frac{deg}{s} \right)^{2})$ |
| 14 | BESS tandem volume ${\boldsymbol{((}\frac{m}{s})}^{2}*\left( \frac{m}{s} \right)^{2}*\left( \frac{deg}{s} \right)^{2})$ |
| 15 | BESS double leg area ${\boldsymbol{((}\frac{m}{s})}^{2}*\left( \frac{m}{s} \right)^{2})$ |
| 16 | BESS tandem area ${\boldsymbol{((}\frac{m}{s})}^{2}*\left( \frac{m}{s} \right)^{2})$ |
| 17 | MDS-UPDRS part 1, total score |
| 18 | MDS-UPDRS part 2, total score |
| 19 | MDS-UPDRS part 2, question 2.13 |
| 20 | MDS-UPDRS part 3, total score* |
| 21 | MDS-UPDRS part 3, question 3.11* |
| 22 | MDS-UPDRS part 4, total score |
| 23 | SMWT distance (meters) |
| 24 | Pre falls within last 6 months (yes/no) |
| 25 | MDT trial average duration (s)* |
| 26 | TUG average velocity $\left( \frac{deg}{s} \right)$ |
| 27 | TUG average duration (s) |
| 28 | TUG average peak velocity $\left( \frac{deg}{s} \right)$ |
| 29 | TUG average turn duration (s) |
| 30 | Gait walk comfortable average velocity* $\boldsymbol{(}\frac{m}{s})$ |
| 31 | Gait walk fast average velocity $\boldsymbol{(}\frac{m}{s})$ |
| 32 | Years since diagnosis* |
| 33 | LEDD total |
| 34 | Non-PD meds, count |
| 35 | PD meds, count |
| 36 | Sex* |
| 37 | Age* (years) |

* Measures employed to compute predictions for the *EMR-retrievable* measures model.

Abbreviations: LEDD, levodopa equivalent daily dose; PD, Parkinson disease; MDS-UPDRS, movement disorders society unified Parkinson’s disease rating scale; MDT, manual dexterity test; BESS, balance error scoring system; PST, processing speed time; SMT, spatial memory test; TMT, trail making test; TUG, timed up and go; s, seconds; EMR, electronic medical records.
